# *Lepidium sativum* Seed Extract-Mediated Synthesis of Zinc Oxide Nanoparticles: Structural, Morphological, Optical, Hemolysis, and Antibacterial Studies

**DOI:** 10.1155/2023/4166128

**Published:** 2023-09-22

**Authors:** Adnan Alnehia, Annas Al-Sharabi, Abdel-Basit Al-Odayni, A. H. Al-Hammadi, Fares H. AL-Ostoot, Waseem Sharaf Saeed, Naaser A. Y. Abduh, Ali Alrahlah

**Affiliations:** ^1^Department of Physics, Faculty of Sciences, Sana'a University, Sana'a, Yemen; ^2^Department of Physics, Faculty of Applied Sciences, Thamar University, Dhamar 87246, Yemen; ^3^Engineer Abdullah Bugshan Research Chair for Dental and Oral Rehabilitation, College of Dentistry, King Saud University, Riyadh 11545, Saudi Arabia; ^4^Department of Biochemistry, Faculty of Education and Sciences, Albaydha University, Albaydha, Yemen; ^5^Department of Chemistry, College of Science, King Saud University, Riyadh 11451, Saudi Arabia

## Abstract

Nanomaterials have unique physicochemical properties compared to their bulk counterparts. Besides, biologically synthesized nanoparticles (NPs) have proven superior to other methods. This work aimed to biosynthesize zinc oxide (ZnO) NPs using an aqueous extract of *Lepidium sativum* seed. The obtained ZnO NPs were characterized by X-ray diffraction, scanning electron microscopy, Fourier transform infrared, and ultraviolet-visible spectroscopy. The *in vitro* antibacterial activity of ZnO NPs against Gram-positive (*S. aureus*) and Gram-negative (*E. coli*) bacteria was assessed using the disk diffusion technique. The hemolytic impact was quantified spectrophotometrically. The results indicated a 24.2 nm crystallite size, a hexagonal structure phase, and a 3.48 eV optical bandgap. Antibacterial studies revealed a dose-dependent response with comparable activity to the standard drug (gentamicin) and higher activity against *S. aureus* than *E. coli*, e.g., the zone of inhibition at 120 mg/mL was 23 ± 1.25 and 16 ± 1.00 mm, respectively. The hemolysis assay showed no potential harm due to ZnO NPs toward red blood cells if utilized in low doses. As a result, it could be concluded that the reported biogenic method for synthesizing ZnO NPs is promising, resulting in hemocompatible NPs and comparable bactericidal agents.

## 1. Introduction

Naturally, zinc oxide (ZnO) nanoparticles (NPs) are an *n*-type semiconductor material with a direct bandgap that exhibits a hexagonal phase and space group of P63mc [1, 2]. It has singular optical characteristics that are significant in new technological fields [3, 4]. At room temperature, ZnO NPs possess extensive excitation binding energy and an optical gap of nearly 3.32 eV [5]. Furthermore, several properties of ZnO NPs make them interesting for a wide range of current applications, including high conductivity, nontoxicity, low cost, environmental friendliness, and thermal stability [6, 7]. The research indicates that ZnO has antibacterial activity against Gram-positive and Gram-negative bacteria owing to its reactive oxygen species (ROS)-producing action, which has been reported to be cytotoxic to bacteria [6, 8]. Furthermore, Zn^2+^ ion releasing from ZnO NPs has fabulous antibacterial activity as previously reported [9].

ZnO is mainly prepared via the green technique and applied for use in different fields like optoelectronic devices and as an additive material in various products such as medical ointments, dental materials, varnish, faience, and glass [10–12]. Various methods can be employed for ZnO NPs preparation, such as (i) physical approaches, including microwave heating, chemical vapor deposition, and thermal evaporation; (ii) chemical routes like coprecipitation process, sonochemical, spray pyrolysis, and hydrothermal procedures; and (iii) biological techniques, including plant extracts (seeds, fruits, leaves, and roots) and microorganisms [6, 11, 13, 14]. Microorganisms such as fungi and bacteria commonly use a variety of purification techniques and additional procedures to preserve cell cultures and intracellular synthesis while making ZnO NPs. Hence, employing the green method for producing ZnO NPs using plant extract is more straightforward than others, including microorganisms [12]. Since water serves as the solvent, the operating output contains no hazardous chemicals, making the method significantly greener and more environmentally friendly [15]. Several active biomolecules derived from plants can be used as reducing and capping agents [16–19]. The NPs made from plants are commonly stable and can be made in various controlled shapes and sizes [20, 21]. Thus, different green plants have been explored as capping agents and stabilizers in the production of NPs [3, 22–26].

In contrast to chemically manufactured analogs, green-synthesized ZnO NPs had a higher antibacterial inhibiting action [10, 13]. Consequently, the biosynthesis technique was preferred for obtaining ZnO NPs with tailorable properties; thus, various biosources were tried, and the results displayed that the bioprepared samples' properties depend on the utilized plant-based extract [9, 15, 27]. To our knowledge, evaluation of the potential of *Lepidium sativum* seed (LSS) extract to prepare ZnO NPs has not been studied so far. *Lepidium sativum* is an herbaceous edible plant that grows in Yemen, Egypt, and West Asia [28]. Since LSS are rich in proteins, fibers, lipids, omega-3, iron, calcium, phosphorus, essential amino acids, and nutrients, they have been used as a medicinal source to treat various diseases. It is reported that the aqueous and alcoholic extracts of LSS are rich with tannins (including tannin acid), phenolics, and flavonoids, which may serve as capping and stabilizing agents in the synthesis of NPs [29].

As a consequence, LSS has become a subject of great interest to researchers since it has no significant side effects or negative impacts [30]. This work was the first to use LSS aqueous extract as a capping agent to produce ZnO NPs. Then, the optical, morphological, structural, antibacterial, and hemolysis characteristics of these phytosynthesized ZnO NPs were assessed.

## 2. Experiments

### 2.1. Materials

Zinc nitrate hexahydrate (Zn (NO_3_)_2_·6H_2_O; BDH; 98%), sodium hydroxide (NaOH; Sigma-Aldrich; 98%), and Gram-positive (*S. aureus*) and Gram-negative (*E. coli*) bacteria were obtained from Taiba Consulting Hospital Laboratories (Dhamar city, Yemen). Mueller–Hinton agar powder and gentamicin (Gnt) were purchased from Sigma-Aldrich. Normal saline (NS) (sodium chloride injection: pH 7.2; made in China) and distilled water (DW) were utilized wherever required.

### 2.2. Preparation of Seed Extract

*Lepidium sativum* seeds were obtained by local sellers in Dhamar city, Yemen, washed with DW, dried at room temperature, and ground using a household blender to a fine powder. Then, 10 g of ground seed powder was suspended in 200 mL of DW and mixed at 25°C with stirring for 30 min. After that, the solution was heated at 60°C for 15 min, brought to room temperature, and then filtered to obtain the target pure extract.

### 2.3. Green Synthesis of ZnO NPs

**Figure 1:**
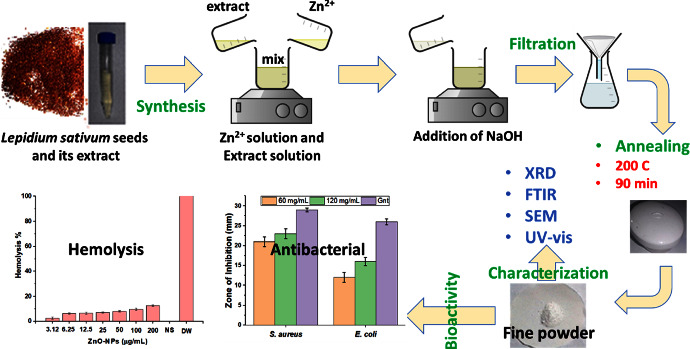
Schematic illustration for the stepwise synthesis protocol of ZnO NPs using *Lepidium sativum* seed extract, characterization, and applications.

In a typical experiment, the zinc nitrate solution was fabricated using 6 g of zinc nitrate salt in 20 mL of DW. The 4 g of sodium hydroxide was dissolved in 20 mL of DW and added dropwise to the zinc nitrate solution. About 3 mL of bioextract was added dropwise, and the reaction mixture was continuously stirred using a magnetic stirrer at room temperature for 75 min. The prepared solution was filtered, and the precipitate was washed twice with DW. The washed precipitate was air-dried for 48 h at room temperature [23, 31]. Finally, the obtained product was annealed at 200°C for 90 min, and the final powder was subjected to various characterizations.  Figure 1 displays the mechanism of preparation, the characterization techniques, and the target bioactivity applications.

### 2.4. Characterization Techniques

The structural properties were analyzed via XRD (CuK*α*, *λ* = 0.15406 nm, 2XD–China) in the 2-theta range 20–80°. Optical properties were investigated via a UV-Vis spectrophotometer (Hitachi, U3900). A scanning electron microscope (SEM, JSM–6360 LV, Japan) was utilized to study the morphological property. FTIR spectroscopy was used to detect functional groups using an FTIR spectrometer (Nicolet iS10, Thermo Scientific, Madison, WI, USA) in the range of 400–4000 cm^−1^ at room temperature.

## 3. Results and Discussion

### 3.1. XRD Analysis

**Figure 2:**
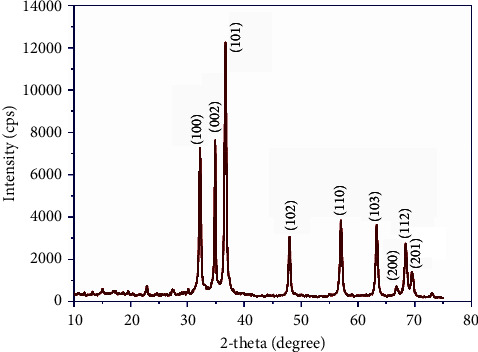
XRD spectra of ZnO NPs.

The XRD pattern of the prepared NPs is utilized to compute crystal lattice indices and crystallite size. Peaks of diffraction are observed at 2-theta values of 32.18°, 34.86°, 36.66°, 47.96°, 57.02°, 63.30°, 68.36°, and 69.51°, corresponding to Miller planes (100), (002), (101), (102), (110), (103), (112), and (201), respectively, as shown in Table 1 and Figure 2. The values were in good agreement with the standard data (card no: 36-1451) [32]. The report illustrates that the prepared sample is hexagonal with space group P63mc [33]. The results confirmed that ZnO NPs were successfully formed. The results agree with previous studies [24, 34]. Furthermore, Figure 2 illustrates the XRD pattern of the prepared NPs with broad diffraction peaks because of the nanocrystalline nature of the synthesized sample. The crystallite size (*D*) was computed via Debye–Scherer's relation *D* = (0.9*λ*/(FWHM)cos *θ*) [35, 36], where *λ* = 0.154 nm, and FWHM and *θ* are the full-width at half maximum and Bragg's angle, respectively [37]. The average crystallite size (*D*_ave_) of the prepared sample was about 24.2 nm. The average dislocation density (*δ*) was computed, and the relation is required to calculate this in the article *δ* = 1/*D*^2^, [27, 35].

The physical parameters like lattice constants, the unit cell volume, and the density of the synthesized sample were investigated using Jade 6 software (MDI). The relations used to compute these parameters have been reported elsewhere [38], and the values are detailed in Table 2.

### 3.2. SEM Analysis

**Figure 3:**
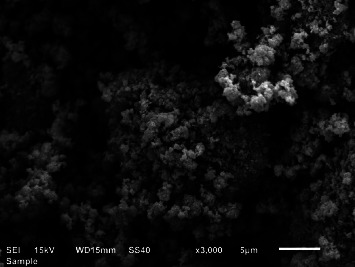
SEM image of the prepared ZnO NPs.

The SEM micrograph of the prepared NPs is shown in Figure 3. The image revealed quasispherical-shaped ZnO nanoparticles, agglomerated into bigger particles, resulting in cluster-type morphology. This could be a result of the polarity as well as the electrostatic attraction between ZnO NPs originating from the material of biological source.

### 3.3. UV-Visible Spectrum Analysis

**Figure 4:**
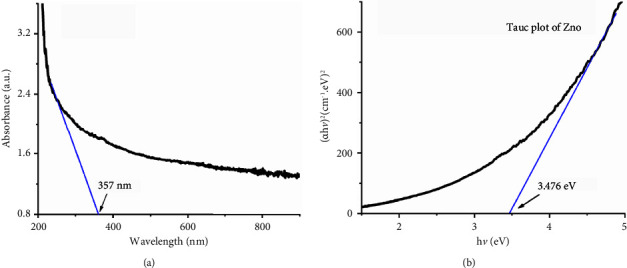
UV-visible spectra and Tauc's plot of ZnO NPs.

The UV-Vis spectrum of ZnO NPs has been recorded in the optical window region at room temperature in the range 200–900 nm, as shown in Figure 4(a). It illustrates that absorption spectra decrease as the wavelength increases. In addition, the spectrum offers an absorption edge at 357 nm. The energy bandgap (*E*_*g*_) is computed via the energy equation: *E*_*g*_=*hc*/*λ*_absorption_ [6], where *h* is Plank's constant, *c* is the speed of light, and *λ*_absorption_ is the absorption wavelength. The computed value of *E*_*g*_ was 3.47 eV. In addition, the optical *E*_*g*_ was calculated by Tauc's relation for a direct allowed transition [39], as shown in Figure 4(b). The evaluated optical *E*_*g*_ is 3.48 eV. Both the computed bandgap and experimental energies are approximately equal. This sort of broad bandgap semiconductors might find applications in, e.g., optoelectronic devices. By comparing the *E*_*g*_ values of ZnO in bulk with the one in the present study, the observed *E*_*g*_ is blue-shifted, possibly due to the quantum confinement effects.

### 3.4. FTIR Spectra Analysis

**Figure 5:**
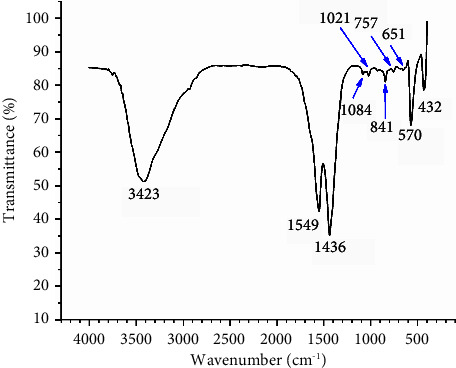
FTIR spectrum of the synthesized ZnO NPs.

FTIR spectroscopy was used to detect functional groups in the compound.  Figure 5 illustrates the FTIR spectrum of the prepared ZnO NPs. The broad band at 3423 cm^−1^ displays the existence of the O-H mode [10]. The absorption peaks at 1400–1600 cm^−1^ represent the C=O stretching mode [25]. The vibrations at 1021 − 570 cm^−1^ denote the existence of C-O-C and C–O, representing traces of esters, ethers and/or carboxylic acids on the green ZnO sample [4]. The band at 432 cm^−1^ is characteristic for ZnO NPs as previously proven by Álvarez-Chimal et al. [40] and Africa et al. [41]. It is important to note that the spectrum of these biosynthesized ZnO NPs is accompanied with peaks for traced organic capping agents from LSS extract, which participated in the reduction and stabilization of the Zn ions, resulting in the production of the target ZnO NPs.

**Figure 6:**
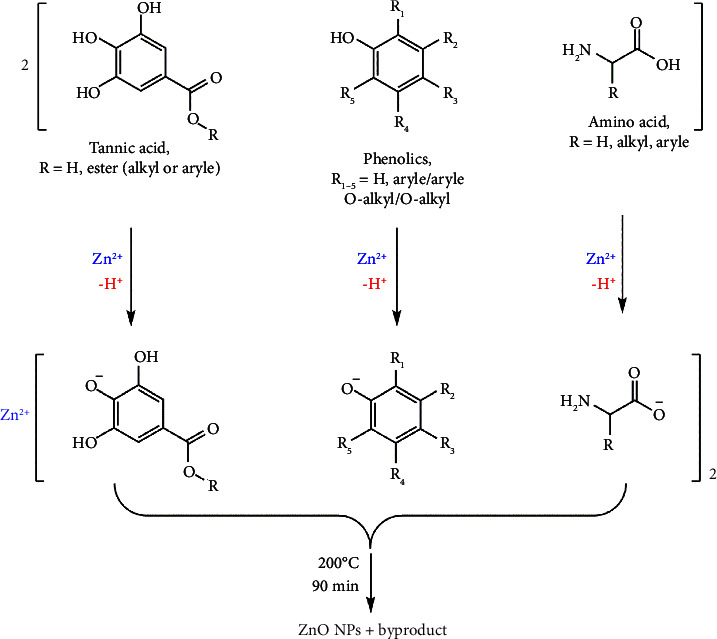
The proposed mechanism for biosynthesis of ZnO NPs using *Lepidium sativum* seed extract-based phytocompounds.

As stated in previous studies, the seed of the *Lepidium sativum* plant contains plenty of tannins, proteins, flavonoids, and some other phenolic derivatives. Therefore, it could be expected that these phytocompounds can serve as great capping and stabilizing agents for Zn^2+^ ion trapping and thus facilitate NPs production [42, 43]. Depending on these major chemicals, a mechanism for the biosynthesis route of ZnO NPs can be predicted [31]. As a result, Figure 6 depicts a proposed mechanism in which the phenolic and carboxylic functional groups of tannins, amino acids, and other phenolic derivatives might be proposed as capping and stabilizing agents for Zn^2+^ ions. By further treating the generated complexes, the final ZnO NPs can be obtained. The interesting efficacy of biosynthesized ZnO NPs via the LSS extract suggests its possible application for synthesizing other valuable metal oxides [18].

### 3.5. Antibacterial Activity

The antibacterial activity of the prepared ZnO NPs was investigated against selected pathogens such as *S. aureus* (Gram-positive) bacteria and *E. coli* (Gram-negative) bacteria using the disc diffusion route [10]. Active bacteria were cultured at 37℃ for 24 h in nutrient broth media, and their optical density was measured using McFarland standard No. 0.5. DW and Gnt were utilized as negative and positive controls, respectively. Different concentrations (60 and 120 mg/mL) of prepared ZnO NPs were loaded on discs and incubated at 36-37℃ for 21 h. The diameter of the inhibition zones was measured after the incubation period had ended.

**Figure 7:**
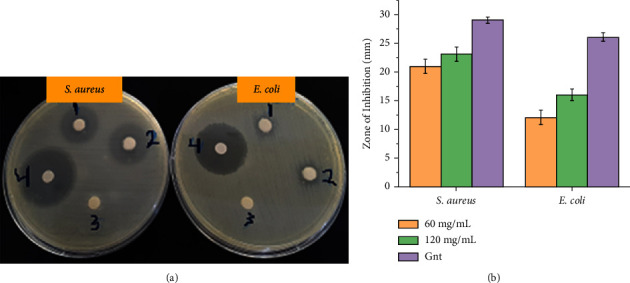
(a) Selected plate images for the antibacterial activity of ZnO NPs against *S. aureus* and *E. coli* bacteria; (1) ZnO NPs 60 mg/mL, (2) ZnO NPs 120 mg/mL, (3) DW (negative control), and (4) gentamicin antibiotics (positive control). (b) Histogram illustration for the corresponding zone of inhibition (ZIO).

The calculated average zone of inhibition (ZOI) (diameters, mm) from two independent experiments is given in Table 3 and Figure 7(a). The results revealed that the antibacterial activity increased with an increase in ZnO NPs concentration (from 60 to 120 mg/mL). At the highest tested concentration (120 mg/mL), the diameter of ZOI for *S. aureus* and *E. coli*have reached 23 and 16 mm, respectively, and was close to the value of the standard drug, Gnt (29 and 26 mm), as shown in Figure 7(b) and Table 3. Hence, the observed high activity of ZnO NPs at higher concentrations is apparently due to a higher number of generated ROS when compared to those formed at a lower concentration. Thus, the inhibitory effect may become more efficient with concentration increase and could generate a higher number of growth inhibitors, like ROS and metal ions, which apparently drive more activity against the tested bacteria. Furthermore, the results indicate higher antibacterial activity for the synthesized ZnO NPs against *S. aureus* (Gram-positive) than *E. coli* (Gram-negative) bacteria. This illustrates that Gram-negative bacteria are less liable to antibacterial potency than Gram-positive bacteria, caused by the impervious and thinner peptidoglycan layer [9, 25, 44].

Table 4 summarizes the antibacterial activity in terms of ZOI of some other ZnO NPs reported in the literature [6, 9, 40, 45–52] for comparison with the present work. As can be seen, the ZOI of the LSS-based biosynthesized ZnO NPs is well positioned in the list or has higher activity than those from the literature. Interestingly, the listed ZnO NPs have shown lesser activity against *E. coli* than *S. aureus* strains, representing Gram-negative and Gram-positive bacteria. However, NPs' bioactivity is commonly influenced by several factors, including their chemistry, morphology, particle size, concentration, and exposure time. Accordingly, the comparison may necessitate additional details; however, for simplicity, the reported ZOI was utilized for comparison, so a clue regarding activity could be gained.

The higher activity of ZnO NPs against Gram-positive bacteria (*S. aureus*) compared to Gram-negative *E. coli* might be a result of their different cell wall structures, which is thicker in Gram-positive and thinner in Gram-negative bacteria. Furthermore, Gram-positive bacteria have only one cytoplasmic membrane with multilayer peptidoglycan polymer, whereas Gram-negative bacteria wall comprises two cell membranes with a thin layer of peptidoglycan. Thus, such variation may explain the difference in susceptibility of ZnO NPs against the examined two bacteria. Nevertheless, ZnO NPs can induce morphological change in bacteria and thus drive functionality loss of the cell.

Though the mechanism of antibacterial activity of ZnO NPs is not well understood and is still controversial, researchers have suggested several pathways; however, ROS formation is the dominant [53, 54]. The antibacterial action is commonly linked with four well-defined mechanisms, which could act individually or simultaneously. Those mechanisms are (i) the direct adhesion onto the surface microorganism cell wall and membrane, (ii) the penetration and release of metal ions into the cell, (iii) the formation of ROS, and (iv) modulation of signal transduction pathways. The final effect would be cell functionality interruption or evenly losses, resulting in cell death [55]. Considerably, generating ROS is one trendy concern and has been a major factor for several mechanisms [55]. Hence, various approaches were proposed to explain the tentative role of ZnO NPs in generating ROS [56]. Typically, the ROS including hydrogen peroxide (H_2_O_2_), hydroxyl radical (OH·), superoxide (O_2_^−^), and Zn^2+^ can react with the bacterial cell wall and intracellular contents of the cell-like proteins, lipids, and carbohydrates, leading to nucleic acids damage, and lastly leads to bacteria death [44]. Apparently, higher ZnO NPs concentrations generate more ROS and thus drive more activity compared with lower ZnO NPs concentrations; this was observed for 60 mg/mL and 120 mg/mL.

### 3.6. Hemolytic Activity

The hemolytic activity of LSS extract-mediated ZnO NPs was estimated against human erythrocytes over a concentration range of 3.12–200 *μ*g/mL. Experimental procedures were performed as described in previous studies [27] with few adjustments. Briefly, 5 mL of blood was taken from a healthy male volunteer (22 years old, O-positive blood group). The blood samples were conveyed into an ethylenediaminetetraacetic acid (EDTA) tube. Then, red blood cells (RBCs) were isolated using a typical procedure described elsewhere [52]. Thus, the EDTA-blood suspension was centrifuged at 4000 rpm for 10 min, decanting the supernatant, and the pellet was adequately washed with 0.9% NS solution. The test erythrocytes suspension was diluted as 2% cells, while check samples of ZnO NPs were prepared as 3.12–200 *μ*g/mL in NS. Experimentally, 0.5 mL of the cell suspension was mixed with 0.5 mL of each test sample and immediately incubated at 37°C for 60 min. After that, solutions were centrifuged at 4000 rpm for 10 min to remove cells depression; the supernatant containing free hemoglobin was photometrically measured at 540 nm. Sterile NS and DW were used as minimal and maximal hemolytic controls and experimentally treated as test samples. The hemolytic percentage was computed based on the following equation [27]:

(1) $$\begin{array}{c} \%\text{ Hemolysis}=\left(\frac{A_{S}-A_{N}}{A_{P}-A_{N}}\right)x\;100\text{,} \end{array}$$

where *A*_*S*_, *A*_*N*_, and *A*_*P*_ represent the absorbance of the sample ZnO NPs, negative control (NS), and positive control (DW), respectively.

**Figure 8:**
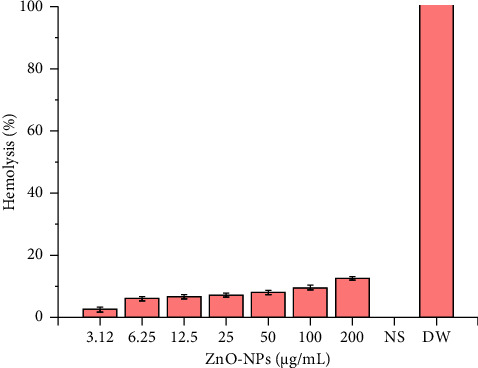
Hemolytic activity of ZnO NPs at different concentrations (3.12–200 *μ*g/mL), NS (normal saline, negative control), and DW (distilled water, positive control).

In order to observe the biosafe nature of ZnO NPs with human RBCs, hemolytic activities were estimated at various concentrations ranging from 3.12 to 200 *μ*g/mL.  Figure 8 displays the average hemolytic activities from two independent experiments. Accordingly, the apparent cytotoxic concentration of ZnO NPs (12.58%) was observed at the higher doses of 200 *μ*g/mL. At the same time, no hemolysis was detected at concentrations less than 6 *μ*g/mL, which strongly agrees with the previous study reported by Muhammad and Ullah [57].

## 4. Conclusion

The ZnO NPs were successfully synthesized using the reported green method. The crystal structure and crystallite sizes were confirmed through XRD. The UV-visible spectrum was employed to determine the absorption edge and compute the optical bandgap. The hemolysis study shows no potential harm due to ZnO NPs to RBCs if used in low doses. Antibacterial results exhibited that the biosynthesized ZnO NPs significantly inhibits the growth of both Gram-positive and Gram-negative bacteria. In addition, the results indicated higher activity of ZnO NPs against *S. aureus* than *E. coli* at all the investigated concentrations. Hence, the data provided the first step in developing new green-synthesized ZnO NPs that have more potent and pharmaceutically acceptable inhibition against both Gram-positive and Gram-negative bacteria that may ultimately be helpful for microbial inhibition.

## Figures and Tables

**Table 1:** Crystallite size, *d*-spacing, and dislocation of ZnO nanoparticles calculated from XRD data.

Sample	(*hkl*)	2–theta (degree)	*d*–spacing (Å)	FWHM (*β*)	Crystallitesize *D* (nm)	Average*D*_ave_ (nm)	Averagedislocation(line/m^2^)*∗*10^15^
ZnO	(100)	32.18	2.779	0.336	24.6	24.2	1.71
	(002)	34.86	2.572	0.252	33.0		
	(101)	36.66	2.449	0.370	22.6		
	(102)	47.96	1.895	0.355	24.5		
	(110)	57.02	1.614	0.401	22.5		
	(103)	63.3	1.468	0.400	23.3		
	(112)	68.36	1.371	0.390	21.4		
	(201)	69.52	1.351	0.449	21.5		

**Table 2:** Geometric parameters ZnO NPs computed from XRD.

Sample	Lattice parameters			(*c*/*a*) ratio	Unit cell volume (Å^3^)	Density (*g*/cm^3^)	Space group
	*a* (Å)	*b* (Å)	*c* (Å)				
ZnO	3.250	3.250	5.207	1.602	47.6	5.68	P63mc

**Table 3:** Antibacterial activity of the prepared ZnO NPs.

Bacteria	ZOI (diameter in mm) ± standard deviation (SD)		
	60 mg/mL	120 mg/mL	Control (gentamicin)
*S. aureus*	21 ± 1.20	23 ± 1.25	29 ± 0.50
*E. coli*	12 ± 1.25	16 ± 1.00	26 ± 0.75

**Table 4:** A comparison of antibacterial activity for the biosynthesized ZnO by *Lepidium sativum* seed extract and the ones reported in literature.

Plant-based extract	Zone of inhibition (mm)		Ref
	*S. aureus*	*E. coli*	
*Aerva lanata* leaf extract	7	8	[45]
*Coleus aromaticus* leaf extract	11	12	[46]
*Dysphania ambrosioides* extract	12	9	[40]
*Eriobotrya japonica* leaf extract	20	17	[47]
*Myrtus communis* L leaf extract	17	—	[48]
*Phoenix roebelenii palm* leaf extract	16	15	[9]
*Punica granatum* leaf extract	—	16	[49]
*Rubus ellipticus* fruits extract	14	8	[50]
*Stachytarpheta jamaicensis*leaf extract	11	0	[6]
*Lepidium sativum* seed extract	23	16	This work

## Data Availability

The data used to support the findings of this study are included within the article.
